# Risk factors for hospitalizations and readmissions among individuals with sickle cell disease: results of a U.S. survey study

**DOI:** 10.1080/16078454.2018.1549801

**Published:** 2019-12

**Authors:** Robert M. Cronin, Jane S. Hankins, Jeannie Byrd, Brandi M. Pernell, Adetola Kassim, Patricia Adams-Graves, Alexis Thompson, Karen Kalinyak, Michael DeBaun, Marsha Treadwell

**Affiliations:** a Department of Biomedical Informatics, Vanderbilt University Medical Center, Nashville, TN, USA; b Department of Internal Medicine, Vanderbilt University Medical Center, Nashville, TN, USA; c Department of Pediatrics, Vanderbilt University Medical Center, Nashville, TN, USA; d Department of Hematology, St Jude Children’s Research Hospital, Memphis, TN, USA; e Department of Pediatrics, Division of Hematology/Oncology, Vanderbilt-Meharry Center for Excellence in Sickle Cell Disease, Vanderbilt University Medical Center, Nashville, TN, USA; f Department of Pediatrics, Division of Hematology, University of Alabama at Birmingham, Birmingham, AL, USA; g Department of Hematology/Oncology, Vanderbilt University Medical Center, Nashville, TN, USA; h Department of General Internal Medicine and Hematology, University of Tennessee Health Science Center, Memphis, TN, USA; i Department of Pediatrics, Department of Medicine, Northwestern University, Chicago, IL, USA; j Division of Hematology in Cancer and Blood Diseases Institute, University of Cincinnati, Cincinnati, OH, USA; k Department of Hematology/Oncology, UCSF Benioff Children’s Hospital Oakland, Oakland, CA, USA

**Keywords:** Health care utilization, clinic visits, vulnerable populations, health care surveys, sickle cell disease, hospital admissions

## Abstract

**Objective:**

Hospital admissions are significant events in the care of individuals with sickle cell disease (SCD) due to associated costs and potential for quality of life compromise.

**Methods:**

This cross-sectional cohort study evaluated risk factors for admissions and readmissions between October 2014 and March 2016 in adults with SCD (*n* = 201) and caregivers of children with SCD (*n* = 330) at six centres across the U.S. Survey items assessed social determinants of health (e.g. educational attainment, difficulty paying bills), depressive symptoms, social support, health literacy, spirituality, missed clinic appointments, and outcomes hospital admissions and 30-day readmissions in the previous year.

**Results:**

A majority of adults (64%) and almost half of children (reported by caregivers: 43%) were admitted, and fewer readmitted (adults: 28%; children: 9%). The most common reason for hospitalization was uncontrolled pain (admission: adults: 84%, children: 69%; readmissions: adults: 83%, children: 69%). Children were less likely to have admissions/readmissions than adults (Admissions: OR: 0.35, 95% CI: [0.23,0.52]); Readmissions: 0.23 [0.13,0.41]). For all participants, missing appointments were associated with admissions (1.66 [1.07, 2.58]) and readmissions (2.68 [1.28, 6.29]), as were depressive symptoms (admissions: 1.36 [1.16,1.59]; readmissions: 1.24 [1.04, 1.49]). In adults, difficulty paying bills was associated with more admissions, (3.11 [1.47,6.62]) readmissions (3.7 [1.76,7.79]), and higher spirituality was associated with fewer readmissions (0.39 [0.18,0.81]).

**Discussion:**

Missing appointments was significantly associated with admissions and readmissions. Findings confirm that age, mental health, financial insecurity, spirituality, and clinic attendance are all modifiable factors that are associated with admissions and readmissions; addressing them could reduce hospitalizations.

## Introduction

Sickle cell disease (SCD) is a disorder of the red blood cells that affects over 100,000 people in the U.S., many of whom are from racial minorities, and who live in low socioeconomic settings [1,2,3,4]. As children are living into adulthood, SCD has become a chronic disease across the lifespan [5]. For the average individual with SCD, medical expenses are over $900,000 by 45 years of age [6]. Hospital admissions and readmissions are very high for individuals with SCD, with approximately 60,000 annual hospital admissions, 90% of admissions for acute pain treatment [4,7,8,9].

Literature about hospitalizations in SCD has described multiple risk factors for admissions and readmissions. Risk factors for readmissions included: age [8,9,10,11], insurance status [8,10], living in low socioeconomic areas [12], lack of outpatient follow up [13,14], other comorbidities like asthma [13], and lack of a primary care provider [15]. Adults are more likely to have admissions and readmissions than children are. The transition period from pediatric to adult care is a vulnerable time period for individuals with SCD with significantly higher admissions than any other period in life [8,16]. Individuals with SCD on Medicaid, which includes about two-thirds of the sickle cell population[4], have higher hospitalizations than those with private insurance [8,15]. In the United Kingdom, Aljuburi et al showed that individuals that lived in socio-economically deprived areas had a higher risk of readmissions. While some literature described that comorbidities like asthma are associated with readmissions [13], others found that pain alone without other sickle cell complications was a risk factor [11]. Finally, not having a primary care provider has also been shown to increase the risk for hospitalizations in SCD[15]. Despite the literature about hospitalizations and readmissions in SCD, studies have never looked at the potential role of missing appointments (preventative sickle cell visits), have mostly been from a single centre, and have not yet explored social and behavioural determinants of health.

This multi-centered study of individuals with SCD across the United States explored factors that affect the admissions and readmissions to the hospital among children and adults with SCD. The study was part of the Mid-South Clinical Data Research Network (CDRN) [17] that included a range of diseases and thousands of enrolled participants. Some questions within the study were derived from the Health Belief Model (HBM) [18] to better appreciate factors that might influence hospitalizations and readmissions. Modifying variables within the HBM may facilitate or hinder positive health actions, which could decrease acute health-care utilization such as hospitalizations and readmissions. We hypothesized that missing appointments, i.e. failing to engage in a positive health action, would be associated with increased hospital admissions and readmissions. Additionally, we explored the association of self-reported measures of mental health, social determinants of health, social support, health literacy, and spirituality with reported hospitalizations and readmissions among individuals with SCD.

## Methods

This project was part of the Mid-South CDRN [17], which was established in 2014 with funding from the Patient-Centered Outcomes Research Institute (PCORI). The 11 CDRN sites in the U.S. have the following collective goals: to engage at minimum 11 million patients across multiple healthcare systems, build infrastructure to share data and build novel informatics tools, and perform comparative effectiveness research and pragmatic clinical trials. The Mid-South CDRN survey tool was designed to obtain uniform information across obesity, coronary heart disease and SCD cohorts. The Institutional Review Boards of the participating sites approved all study procedures and informed consent was obtained from all participants.

### Setting and procedure

Between October 2014 and March 2016, we surveyed a convenience sample of adults with SCD (age ≥18 years) and caregivers of children with SCD (patients age < 18 years). Surveys were completed at sites at six sickle cell centres across the U.S.: Cincinnati Children’s Hospital Medical Center, Children’s Hospital of Chicago, University of Tennessee Health Science Center, St. Jude Children’s Research Hospital, Vanderbilt University Medical Center, and UCSF Benioff Children’s Hospital Oakland. Eligibility criteria included individuals who could speak and read English, had a diagnosis of SCD (of any phenotype) or were caregivers of children with SCD, and had care delivered at one of the six participating centers. Individuals with SCD and their caregivers were recruited with flyers in clinics and by their health care providers during clinic visits. The surveys were administered via computer tablet, or paper-and-pencil if a tablet was not available. Members of the research team were present if participants required assistance. Participants received a gift card upon completion of the survey. The targeted sample size was 450 for this survey research, for a margin of error of 5% and a confidence interval of 95%, with expectations for numbers enrolled at each study site based on the size of their patient population.

### Surveys

The surveys were designed with the input of a variety of stakeholders including researchers, healthcare providers, social workers, psychologists and individuals with SCD. The final set of question domains were derived based on stakeholder input and included sociodemo-graphic variables, depressive symptoms, social support, health literacy and spirituality.

Adults with SCD and caregivers of children reported on missed clinic appointments within the past year. The outcome measures included any hospitalizations or readmissions, defined as being admitted to the hospital twice in a 30-day period. Individuals with SCD or caregivers reported on admissions or readmissions. Survey participants provided reasons for hospitalizations and readmissions.

Social determinants of health included sex, race, ethnicity, educational attainment, difficulty paying bills, and marital status. We combined some categories of survey responses for ease of interpretation within the regression analyses. Caregivers responded about themselves for the educational attainment, difficulty paying bills, and marital status, and answered about their child for the other questions.

Depressive symptoms were measured by the Patient Health Questionnaire (PHQ-2 [19]), a validated two-item screening for the frequency of depressed mood and anhedonia over the past two weeks. PHQ-2 scores range from 0 (not at all) to 6 (nearly every day), with a score of 3 suggesting the need for further evaluation of depressive disorder [19]. Depressive symptoms were answered by caregivers about their children with SCD.

Participants rated their social supports using the ENRICHD (Enhancing Recovery in Coronary Heart Disease) Social Support Inventory (ESSI [20]). Participants rated whether they had someone to whom they felt close, could give them advice, show love and affection, and provide emotional support at difficult times on a scale from “None of the time” (1) to “All of the time” (5). Low support on the ENRICHD ESSI has been defined as 2 or more items <=2, or 2 or more items <=3 and an adjusted overall score <=18 [21]. Caregivers responded to this measure in relation to their child’s social support.

Health literacy, or the ability to obtain, read, understand and use healthcare information to make appropriate health decisions, is an important factor that can lend to positive health actions such as keeping appointments [22]. Health literacy was evaluated using the Brief Health Literacy Screening [23,24]. Inadequate health literacy can be determined from one or a combination of all three of these questions [23,24]. Responses of “somewhat” or better for the question “How confident are you filling out medical forms by yourself?” has been used to define “good” health literacy [23]. Caregivers responded about their health literacy, not their child’s.

Spirituality, an emerging focus of study in relation to health behaviours [25] particularly for African Americans [26] was rated by participants from very (1) to not at all (4). Participants rated how spiritual they considered themselves to be using a single item “how spiritual or religious do you consider yourself (or your child) to be,” from very (1) to not at all (4). Based on the distribution of the responses and for ease of analysis, we dichotomized the variable into “very” spiritual (option 1) and “not very” spiritual (options 2–4). Caregivers responded about their child’s spirituality.

### Statistical analysis

Study data were collected, deidentified, and managed using the REDCap electronic data capture tools hosted at Vanderbilt University [27]. Surveys were excluded for missing data about age, site, sex, missed appointments, admissions, or readmissions. Descriptive statistics were used to summarize demographics, social and behavioural determinants of health, and other questions. Means and interquartile ranges were used for continuous variables, and proportions for categorical variables.

We created logistic regression models for the outcome measure of admissions and readmissions using potential risk factors i.e. social determinants of health (gender, age, education level, ability to pay bills), depressive symptoms, health literacy, social support, and spirituality. Initially models were created for all participants, but given that adults and children with SCD have important differences in admissions and readmissions, we created a variable that dichotomized adults and children. As adult and pediatric models of care can be important predictors for hospitalizations, we performed a supplementary analysis based on the type of model of care or clinic (pediatric or adult) a participant came from (Supplemental Tables 1–6). Since the survey was anonymous, we cannot definitively determine which clinic participants were recruited from in mixed clinical sites (e.g. Vanderbilt), therefore, we used a surrogate of 18 years of age to differentiate pediatric and adult models of care. Statistically significant differences in our regression for adults compared with children led to two new models for adults and children to evaluate differences. Analyses were performed in R version 3.2.2, and p-values were considered significant if < 0.05 [28].

## Results

### Demographics

A total of 573 individuals with SCD (adults and caregivers of children with SCD) completed the surveys at a single clinic visit. After excluding surveys with missing data, our final sample for analysis included 201 adults with SCD and 330 caregivers of children with SCD (*n* = 531). We oversampled our population to accommodate missing data and exceeded our projected sample size of 450. Table 1 shows the distribution of adults and pediatric patients.

### A majority of participants missed appointments because of forgetting, had hospitalizations and readmissions because of pain, but differed on reasons depending on whether they were adults or caregivers of children

The majority of the adult and caregivers of children reported missing an appointment over the last year (87% and 65% respectively). The most common reason was forgetting: 31% among adults and 26% among caregivers. The second most common reason was appointments not scheduled at a convenient time for adults (25%) and not having transportation to get to the appointment for caregivers of children (23%).

majority of adults (64%) and almost half of children as reported by caregivers (43%) were admitted, and 28% of adults and 9% of caregivers of children reported being readmitted over the prior year (Table 2). The most common reason for admissions in adults and children was uncontrolled pain (adults: 84%, children as reported by caregivers: 69%), and the second most common reason was to get fluids or a blood transfusion in both children and adults, but the third most common reason was a fever as reported by caregivers in children (55%) and that their medication was not working in adults (44%). The most common reason for readmission was uncontrolled pain in adults and children (adults: 83%, children as reported by caregivers: 69%). The second most common reason was that the medication was not working in adults (59%) and a fever as reported by caregivers in children (48%). An inability to get medications was a much higher reason for admission and readmission in children and adults (admission: 29% vs 5%, readmission: 38% vs 7%). Over half of the adults (57%) and nearly one third of the caregivers of children (31%) thought the patient was not healthy enough to leave the hospital during the first stay. This may indicate premature discharge in some cases.

### Missing appointments was associated with increased hospitalizations and readmissions

For all participants, missed appointments was significantly associated with increased admissions to the hospital (OR: 1.66, 95% CI: [1.07, 2.58]) and increased readmissions to the hospital (OR: 2.68, 95% CI: [1.28, 6.29]) (Table 3). When looking at adults and children separately, adults who missed appointments were sig-nificantly more likely to have admissions (OR: 3.15, 95% CI: [1.09, 9.57]) and readmissions (OR: 8.16, 95% CI: [1.23, 347]); however, children and caregivers who missed appointments did not have significantly more admissions or readmissions.

### Participants had difficulty paying bills, had moderate rates of depression

Forty-five percent of the total sample reported it was “somewhat” to “very difficult” to pay monthly bills and 42% of the total sample rated themselves as “very” spiritual or religious. The mean score on the PHQ-2 for depression in adults (1.46 ± 1.55) was higher than caregivers reported for children (0.84 ± 1.26), with *n* = 49 (23.2%) adults and *n* = 47 (14.2%) of caregivers of children reported of scores of 3 and above on the PHQ-2 (Table 4). Most participants rated social support (85%) and health literacy as “good” (75%).

### Being an adult, having more depressive symptoms, and difficulty paying bills were associated with more admissions

Children (as reported by caregivers) were less likely to be hospitalized than adults (Odds Ratio (OR) = 0.35; 95% Confidence Interval (CI) = [0.23, 0.52]) (Table 5). In all individuals with SCD, more depressive symptoms (OR = 1.36; 95% CI = [1.16, 1.59]) was associated with more admissions. In adults and in children, depressive symptoms (adults: OR = 1.34; 95% CI = [1.04, 1.72]; children as reported by caregivers: OR = 1.32; 95% CI = [1.07, 1.63]) associated with more admissions. However, difficulty paying bills was only significantly associated with hospitalizations in adults (OR = 3.11; 95% CI = [1.47, 6.62]). Our supplemental analysis (Supplemental Tables 2–6) demonstrated similar findings as the primary analysis, except some associations were stronger, such as difficulty paying bills with hospitalizations in adults (OR = 4.43; 95% CI = [1.69, 11.58]), as well as another association becoming significant with older adults being less likely to have hospitalizations in the prior year (OR = 0.96; 95% CI = [0.93,0.99]). For each year of age in adults, there was a 4% lower chance of being admitted in the previous 12 months.

### Depressive symptoms, difficulty paying bills, being an adult, and being less spiritual were all significantly associated with readmissions

Children as reported by caregivers were much less likely to be readmitted than adults (Odds Ratio (OR) = 0.23; 95% Confidence Interval (CI) = [0.13, 0.41]) (Table 5). In all individuals with SCD, depressive symptoms (OR = 1.24; 95% CI = [1.04, 1.49]), difficulty paying bills (OR = 2.4; 95% CI = [1.36, 4.24]) were associated with readmissions and being very spiritual (OR = 0.57; 95% CI = [0.33, 0.99]) was associated with fewer readmissions. In the adult model, ability to pay bills (OR = 3.7; 95% CI = [1.76, 7.79]) and spirituality (OR = 0.39; 95% CI = [0.18, 0.81]) remained significant, but nothing remained significant in the pediatric model. Our supplemental analysis showed similar results as the primary analysis, and older age of the adult was associated with less readmissions (OR = 0.96, 95% CI = [0.92, 0.99]).

## Discussion

Our manuscript is one of the first to describe associations between missed appointments and hospitalizations for a sample including both adults and children with SCD from multiple sickle cell centres across the U.S., as well as considering social and behavioural risk factors for admissions and readmissions among individuals with SCD. Missing and forgetting appointments were significantly associated with more hospitalizations and readmissions, indicating the importance of outpatient care in this population. There were several important risk factors that were associated with admissions including more depressive symptoms and more difficulty paying bills. Readmissions were associated with these same variables, but also with less spirituality. In our supplementary analysis, in adults with SCD seen in adult clinics, younger age was associated with more hospitalizations and read-missions, potentially emphasizing the importance of the critical period after transition from pediatric to adult models of care. With acute healthcare utilization being costly and disruptive for individuals with SCD, these variables demonstrate potential targets for interventions that could help improve the care for individuals with SCD.

Missing appointments were significantly associated with more adult hospitalizations and readmissions. Appointments are a very important component of care in SCD, as lack of outpatient follow up and access to primary care providers (PCPs) have been implicated in more hospitalizations [13,14,15]. In related work, we demonstrated multiple risk factors for missed appointments, which could be targeted to improve attendance at clinic appointments and help decrease hospitalizations [29]. Missed appointments were not significantly associated with hospitalizations and readmissions in children as reported by caregivers, which could be related to the smaller sample size of readmitted children, different reasons for hospitalizations, children having fewer complications than adults, or the larger role PCPs play in the pediatric population. The overall severity of the disease may generate a particular patient profile in which missing appointments are only one part of the overall picture.

Adults and children with SCD have different risk factors for admissions and readmissions. Being an adult, by itself, was highly associated with more admissions and readmissions, which has been demonstrated with complications of aging in prior literature [8,9,10,11,15]. The transition period is a very critical time for individuals with SCD with more hospitalizations occurring in the young adult population. Our supplementary analysis findings emphasize young adulthood as a potential risk factor for both admissions and readmissions once transitioned to an adult model of care, given that those seen in adult clinics who were younger in age had more hospitalizations. More depressive symptoms were significant in our adult models for admissions and readmissions, as well as in the pediatric model for admissions. Depression has been shown to be a significant contributor to hospital admissions in prior literature [30]; however, depression has not been previously shown to be important in readmissions. More difficulty paying bills, a likely surrogate for lower socioeconomic status, was an important factor in the adult admission and readmission model. Lower socioeconomic status has been shown to be an important factor in hospitalizations [12]. Spirituality also played a significant role in the readmissions of the hospital population. The role of spirituality has previously been shown in hospitalizations in adults with SCD [31,32,33], but we demonstrate that it may have significant implications in readmissions across a large cohort across the nation. This study replicates many of the findings seen in other literature in a large national cohort and adds additional potential intervention targets that could help decrease readmissions within the SCD population.

Reasons for hospitalizations in children as reported by caregivers were different than in adults, with fever being a more likely reason in children than adults. These differences could explain the loss of significance, and that the reason for hospitalization may be associated with different risk factors (e.g. a fever may not be associated with lower socioeconomic status, but not having medications would). The inability to get medication is a reported reason that needs further exploration of the exact medications prescribed, financial ability to pay for them, transportation issues to obtain them, and who prescribed them, all potentially important factors as to why the inability to get a medication was mentioned as a reason for hospitalizations. Further research into the factors that lead to different reasons for hospitalizations and readmissions is needed. PCPs are an essential part of the care of individuals with SCD, and not having a PCP has been shown to be associated with higher rates of hospitalizations and readmissions [15].

### Limitations

Several weaknesses or limitations caution interpretation of these results. First, we used self-report surveys, which can lead to recall bias. While hospitalizations are a significant event and individuals are more likely to remember the disruptive nature of these events, there is the possibility that they may not recall them accurately. Future research evaluating the accuracy of self-reported admissions and readmissions as compared to electronic health record data could help support the current findings. We also evaluated readmissions by asking if individuals were admitted to the hospital twice in one month, which may not be the best metric of quality of care in SCD, however, about 50% of participants said yes to the question “I (or my child) was not healthy enough to leave the hospital during the first stay,” which may indicate that the participant or family member felt they went home pre-maturely. Readmissions within 30 days may not have the same implied meaning as in heart failure[34], as this may be indicative of a more severely affected person with SCD. However, having to come into the hospital and back into the hospital multiple times a month constitutes its own problem. More work is needed to determine the best metric of quality of care in SCD. In addition, we are unable to identify specific hospitals where the admissions and readmissions occurred. Many of our sites do have hospitals affiliated with the outpatient clinics (Supplemental Table 1), but an area of future research could determine if there are differences in individuals who have admissions at hospitals not affiliated with sickle cell centres.

Second, while we recruited from sickle cell centres across the U.S., responses may differ in other areas of the U.S. that were not sampled. In particular, the high percentage of participants contributed by one site (e.g. St. Jude for children, and Vanderbilt for adults), and the fact that we recruited fewer adults than children could limit the generalizability to the entirety of the adult population of SCD, especially adults. We also recruited a convenience sample of participants, which may lead to selection bias, however participants were sequentially approached, without any selection for disease severity or social factors, potentially reducing selection bias. Third, we cannot guarantee if the caregivers were answering the questions about them-selves or the child patient. However, coordinators who administered the surveys did not report that there seemed to be confusion among caregivers regarding who the questions were concerned with. Fourth, spirituality was measured with a single item, and while this item is part of a validated instrument [35], further studies with more detailed instruments could further evaluate spirituality’s relationship, as a potential coping strategy, to readmissions. Fifth, we elucidated associations with hospitalizations and read-missions, and cannot imply causation. Further research is needed to demonstrate if the factors that emerged in our analyses cause hospitalizations or readmissions. Finally, missing response data and other factors not collected (e.g. insurance coverage) that could also contribute to admissions and readmissions may have reduced our ability to identify important factors. However, the amount of missing data was relatively small and factors investigated were significant in our models. Additional factors, including the number of hospitalizations in the year prior to study, an inability to get medications, the quality of the outpatient care received, and treatments for SCD such as transfusion therapy would allow us to paint a more complete picture of the severity of the person’s SCD. These factors are important potential risk factors that could predict hospitalizations and need to be evaluated in future research.

## Conclusions

Our results highlighted a number of modifiable risk factors associated with hospital admissions and read-missions for individuals with SCD across the U.S. We found that missing appointments in the clinic is potentially an important factor in admissions and readmissions for these individuals, in addition to being a younger adult seen in the adult model of care, more depressive symptoms, and more difficulty paying bills. More work is needed to demonstrate whether spirituality, which has been shown to be an important coping strategy in SCD [33], can be tapped as a beneficial approach to reduce admissions and readmissions. Interventions to improve clinic appointment keeping, address depressive symptoms, and improve the transition from the pediatric to adult model of care could potentially reduce admissions and readmissions for this population. Understanding factors that influence admissions and readmissions is important when considering effective strategies to improve the care of individuals with SCD.

## Supplementary Material

Supplementary Tables 1-6

## Figures and Tables

**Table 1.** Socio-demographics for adult participants with sickle cell disease.

Variable		Adults (*N* = 201)	Children^a^ (*N* = 330)	Combined (*N* = 531)
Age	Years (SD, Range)	26.0 (22.0–35.0)	10.0 (6.0–14.0)	14.0 (8.0–24.0)
Sex	Male	85 (40.3%)	160 (48.3%)	245 (45.2%)
	Female	116 (55.0%)	170 (51.4%)	286 (52.8%)
Race/Ethnicity	Black, African American, African, or Afro-Caribbean	193 (91.5%)	323 (97.6%)	516 (95.2%)
	Hispanic, Latino, or Spanish origin	5 (2.4%)	5 (1.5%)	10 (1.8%)
	Some other race or origin	10 (4.7%)	9 (2.7%)	19 (3.5%)
Highest degree or level of school completed	High school graduate or less	85 (40.3%)	133 (40.2%)	218 (40.2%)
	Some college or beyond	112 (53.1%)	126 (38.1%)	238 (43.9%)
Household size	Median (Range)	4 (1–14)	3 (1–8)	4 (1–14)
Marital status	Married/Living together	50 (23.7%)	110 (33.2%)	160 (29.5%)
	Unmarried	151 (71.6%)	220 (66.5%)	371 (68.5%)
Spirituality/Religiosity	Very	75 (35.5%)	151 (45.6%)	226 (41.7%)
	Fairly	87 (41.2%)	102 (30.8%)	189 (34.9%)
	Slightly/Not at All	39 (18.5%)	60 (18.1%)	99 (18.3%)
Difficulty paying monthly bills	Not very/Not at all	114 (54.0%)	176 (53.2%)	290 (53.5%)
	Somewhat/Very	87 (41.2%)	154 (46.5%)	241 (44.5%)
Site	Midwest region:			
	Cincinnati	11 (5.2%)	40 (12.1%)	51 (9.4%)
	Chicago	17 (8.1%)	84 (25.4%)	101 (18.6%)
	Western region:			
	Oakland	44 (20.9%)	0 (0.0%)	44 (8.1%)
	Mid-South region:			
	St. Jude	6 (2.8%)	155 (46.8%)	161 (29.7%)
	UTHSC^c^	40 (19.0%)	0 (0.0%)	40 (7.4%)
	Vanderbilt	83 (39.3%)	51 (15.4%)	134 (24.7%)

a Caregivers were asked to report for their children under 18 years.

b Percentages may not add up to 100% because of missing data.

c UTHSC = University of Tennessee Health Science Center.

**Table 2.** Questions about admissions, readmissions, and appointment keeping (*n* = 531).

Question	Response	Adults (*N* = 201)	Children^a^ (*N* = 330)	Combined (*N* = 531)
**Admissions**				
Have you (or your child) been admitted to the hospital withinthe last year?	No	66 (31.3%)	189 (57.1%)	255 (47.0%)
	Yes	135 (64.0%)	141 (42.6%)	276 (50.9%)
I was unable to get the medication(s) I (or my child) needed.	No	91 (67.4%)	134 (95%)	225 (81.5%)
	Yes	39 (28.9%)	7 (5%)	46 (16.7%)
I did not feel that my (or my childs) medication was working.	No	71 (52.6%)	101 (71.6%)	172 (62.3%)
	Yes	59 (43.7%)	40 (28.4%)	99 (35.9%)
I did not have a good understanding of how often and how much of each medication I (or my child) needed.	No	117 (86.7%)	135 (95.7%)	252 (91.3%)
	Yes	12 (8.9%)	6 (4.3%)	18 (6.5%)
I did not have a good understanding of the major side effects of my (or my childs) medications.	No	120 (88.9%)	132 (93.6%)	252 (91.3%)
	Yes	11 (8.1%)	9 (6.4%)	20 (7.2%)
My (or my childs) pain was not able to be controlled at home.	No	21 (15.6%)	44 (31.2%)	65 (23.6%)
	Yes	113 (83.7%)	97 (68.8%)	210 (76.1%)
I did not have all the information I needed to take care of my (or my childs) illness at home.	No	113 (83.7%)	130 (92.2%)	243 (88%)
	Yes	17 (12.6%)	11 (7.8%)	28 (10.1%)
I did not understand which warning signs and symptoms meant	No	114 (84.4%)	131 (92.9%)	245 (88.8%)
I should call my (or my childs) healthcare provider.	Yes	16 (11.9%)	10 (7.1%)	26 (9.4%)
I (or my child) had an illness unrelated to their sickle cell disease (e.g. asthma).	No	79 (58.5%)	102 (72.3%)	181 (65.6%)
	Yes	50 (37%)	39 (27.7%)	89 (32.2%)
I (or my child) needed to get fluids or blood transfusion	No	40 (29.6%)	60 (42.6%)	100 (36.2%)
	Yes	90 (66.7%)	81 (57.4%)	171 (62%)
I (or my child) had a fever	No	80 (59.3%)	64(45.4%)	144 (52.2%)
	Yes	51 (37.8%)	77 (54.6%)	128 (46.4%)
**Readmissions**				
Have you (or your child) been admitted to the hospital twice in the same month within the last year?	No	143 (67.8%)	301 (90.9%)	444 (81.9%)
	Yes	58 (27.5%)	29 (8.8%)	87 (16.1%)
I was unable to get the medication(s) I (or my child) needed.	No	30 (51.7%)	27 (93.1%)	57 (65.5%)
	Yes	22 (37.9%)	2 (6.9%)	24 (27.6%)
I did not feel that my (or my childs) medication was working.	No	22 (37.9%)	20 (69%)	42 (48.3%)
	Yes	34 (58.6%)	9 (31%)	43 (49.4%)
I did not have a good understanding of how often and how much of each medication I (or my child) needed.	No	48 (82.8%)	28 (96.6%)	76 (87.4%)
	Yes	4 (6.9%)	1 (3.4%)	5 (5.7%)
I did not have a good understanding of the major side effects of my (or my childs) medications.	No	46 (79.3%)	26 (89.7%)	72 (82.8%)
	Yes	5 (8.6%)	3 (10.3%)	8 (9.2%)
My (or my childs) pain was not able to be controlled at home.	No	8 (13.8%)	9 (31%)	17 (19.5%)
	Yes	48 (82.8%)	20 (69%)	68 (78.2%)
I did not have all the information I needed to manage my (or my childs) illness at home.	No	43 (74.1%)	24 (82.8%)	67 (77%)
	Yes	10 (17.2%)	5 (17.2%)	15 (17.2%)
I did not understand which warning signs and symptoms meant	No	45 (77.6%)	25 (86.2%)	70 (80.5%)
I should call my (or my childs) healthcare provider.	Yes	7 (12.1%)	4 (13.8%)	11 (12.6%)
I (or my child) had an illness unrelated to their sickle cell disease (e.g. asthma).	No	33 (56.9%)	23 (79.3%)	56 (64.4%)
	Yes	19 (32.8%)	6 (20.7%)	25 (28.7%)
I (or my child) was not healthy enough to leave the hospital during the first stay.	No	20 (34.5%)	20 (69%)	40 (46%)
	Yes	33 (56.9%)	9 (31%)	42 (48.3%)
I (or my child) had a fever	No	29 (50%)	14 (48.3%)	43 (49.4%)
	Yes	24 (41.4%)	14 (48.3%)	38 (43.7%)
**Missed appointments**				
Have you missed an appointment for any reason over the past year?	No	26 (12.3%)	114 (34.4%)	140 (25.8%)
	Yes	183 (86.7%)	216 (65.3%)	399 (73.6%)
Reasons you missed an appointment	I forgot I had an appointment	65 (30.8%)	56 (25.9%)	121 (30.3%)
	The appointment was at a time that didn’t work for me	53 (25.1%)	42 (19.4%)	95 (23.8%)
	My health impacted my ability to make the appointment	40 (19.0%)	9 (4.2%)	49 (12.3%)
	I did not know I had an appointment	39 (18.5%)	22 (10.2%)	61 (15.3%)
	I didn’t have a way to get to the appointment	44 (20.9%)	50 (23.1%)	94 (23.6%)

a Caregivers were asked to report for their children under 18 years.

b Percentages may not add up to 100% because of missing data.

**Table 3.** Relation between reasons for missing clinic appointments and hospital admissions and readmissions.

Hospital admission in the past year		
**Combined**		
Have you missed an appointment for any reason	No	Yes
No admission	66 (57.9%)	189 (45.3%)
Admission	48 (42.1%)	228 (54.7%)
*P*-value	0.02	
OR (95% CI)	1.66 (1.07–2.58)	
**Adults**		
Have you missed an appointment for any reason	No	Yes
No admission	11 (57.9%)	55 (30.2%)
Admission	8 (42.1%)	127 (69.8%)
*P*-value	0.02	
OR (95% CI)	3.15 (1.09–9.57)	
**Children**^a^		
Have you missed an appointment for any reason	No	Yes
No admission	55 (57.9%)	134 (57%)
Admission	40 (42.1%)	101 (43%)
*P*-value	0.903	
OR (95% CI)	1.04 (0.62–1.73)	

Readmission in the past year		

**Combined**		
Have you missed an appointment for any reason	No	Yes
No readmission	105 (92.1%)	339 (81.3%)
Readmission	9 (7.9%)	78 (18.7%)
*P*-value	0.004	
OR (95% CI)	2.68 (1.28–6.29)	
**Adults**		
Have you missed an appointment for any reason	No	Yes
No readmission	18 (94.7%)	125 (68.7%)
Readmission	1 (5.3%)	57 (31.3%)
*P*-value	0.016	
OR (95% CI)	8.16 (1.23–347.37)	
**Children**^a^		
Have you missed an appointment for any reason	No	Yes
No readmission	87 (91.6%)	214 (91.1%)
Readmission	8 (8.4%)	21 (8.9%)
*P*-value	1	
OR (95% CI)	1.07 (0.43–2.90)	

a Caregivers were asked to report for their children under 18 years.

**Table 4.** Scores on standardized measures for the adult participants (*n* = 531) with sickle cell disease.

Measure		Adults (*N* = 201)	Children^a^ (*N* = 330)	Combined (*N* = 531)
Patient Health		1.47 (1.56)	0.84 (1.26)	1.08 (1.41)
Questionnaire (PHQ-2; mean/SD)				
ENRICHD Social	Poor	44 (20.9%)	31 (9.4%)	75 (13.8%)
Support Instrument (ESSI) (n/%)	Good	157 (74.4%)	299 (90.3%)	456 (84.1%)
Brief Health Literacy	Poor	58 (27.5%)	75 (22.7%)	133 (24.5%)
Screening (n/%)	Good	143 (67.8%)	255 (77.0%)	398 (73.4%)

a Caregivers were asked to report for their children under 18 years.

b Percentages may not add up to 100% because of missing data.

**Table 5.** Logistic regression model: risk factors for hospitalization and readmissions.

Hospitalizations				
*Combined Model*	(*n* = 531)			
Variable		Odds Ratio	95% CI	Pr(>|z|)
(Intercept)		1.1	(0.49,2.46)	0.815
Age Group	Pediatrics (<18 y/o)	0.35	(0.23,0.52)	<0.001*
Sex	Female	1.14	(0.77,1.7)	0.509
Education:	Some college or more	1.22	(0.8,1.85)	0.348
PHQ score		1.36	(1.16,1.59)	<0.001*
Ability to pay bills	Very or somewhat difficult	1.46	(0.97,2.21)	0.073†
Literacy	High	0.87	(0.53,1.43)	0.592
Spirituality	Very spiritual	0.78	(0.51,1.17)	0.224
Social Support	High	1.21	(0.66,2.22)	0.539
*Adult Model*	(*n* = 201)			
Variable		Odds Ratio	95% CI	Pr(>|z|)
(Intercept)		0.65	(0.14,2.96)	0.579
Age		0.99	(0.97,1.02)	0.615
Sex	Female	0.94	(0.49,1.82)	0.859
Education:	Some college or more	1.55	(0.79,3.04)	0.199
PHQ score		1.34	(1.04,1.72)	0.021*
Ability to pay bills	Very or somewhat difficult	3.11	(1.47,6.62)	0.003*
Literacy	High	1.67	(0.77,3.64)	0.193
Spirituality	Very spiritual	0.8	(0.39,1.63)	0.536
Social Support	High	1.2	(0.49,2.93)	0.691
*Model about Children*	(*n* = 330)			
Variable		Odds Ratio	95% CI	Pr(>|z|)
(Intercept)		0.56	(0.16,1.95)	0.365
Age		1	(0.95,1.06)	0.889
Sex	Female	1.14	(0.68,1.92)	0.628
Education:	Some college or more	1.07	(0.62,1.85)	0.8
PHQ score		1.32	(1.07,1.63)	0.011*
Ability to pay bills	Very or somewhat difficult	1.04	(0.61,1.76)	0.881
Literacy	High	0.6	(0.31,1.17)	0.135
Spirituality	Very spiritual	0.78	(0.46,1.33)	0.368
Social Support	High	1.37	(0.56,3.36)	0.493
**Readmissions**				
*Combined Model*	(*n* = 531)			
Variable		Odds Ratio	95% CI	Pr(>|z|)
(Intercept)		0.26	(0.09,0.72)	0.009
Age Group	Pediatrics (<18 y/o)	0.23	(0.13,0.41)	<0.001*
Sex	Female	1.37	(0.79,2.37)	0.268
Education:	Some college or more	1.05	(0.6,1.84)	0.868
PHQ score		1.24	(1.04,1.49)	0.019*
Ability to pay bills	Very or somewhat difficult	2.4	(1.36,4.24)	0.002*
Literacy	High	1.25	(0.65,2.41)	0.504
Spirituality	Very spiritual	0.57	(0.33,0.99)	0.046*
Social Support	High	0.63	(0.32,1.28)	0.204
*Adult Model*	(*n* = 201)			
Variable		Odds Ratio	95% CI	Pr(>|z|)
(Intercept)		0.57	(0.11,2.87)	0.496
Age		0.98	(0.95,1.01)	0.164
Sex	Female	1.16	(0.56,2.39)	0.687
Education:	Some college or more	1.31	(0.64,2.7)	0.458
PHQ score		1.18	(0.94,1.49)	0.145
Ability to pay bills	Very or somewhat difficult	3.7	(1.76,7.79)	0.001*
Literacy	High	1.35	(0.6,3.04)	0.475
Spirituality	Very spiritual	0.39	(0.18,0.81)	0.012*
Social Support	High	0.53	(0.23,1.24)	0.146
*Model about Children*	(*n* = 330)			
Variable		Odds Ratio	95% CI	Pr(>|z|)
(Intercept)		0.02	(0,0.24)	0.002
Age		1.04	(0.94,1.16)	0.432
Sex	Female	1.41	(0.55,3.66)	0.477
Education:	Some college or more	0.84	(0.32,2.24)	0.729
PHQ score		1.33	(0.96,1.84)	0.083†
Ability to pay bills	Very or somewhat difficult	1.19	(0.47,3.04)	0.711
Literacy	High	1.29	(0.38,4.36)	0.677
Spirituality	Very spiritual	0.97	(0.37,2.5)	0.944
Social Support	High	1.23	(0.25,6.13)	0.798

Note: UTHSC: University of Tennessee Health Science Center; CHO: Children’s Hospital Oakland; CCHMC: Cincinnati Children’s Hospital Medical Center.

* *p* < 0.05.

† *p* < 0.1.
