# Supplementary material for: Risk factors for hospitalizations and readmissions among individuals with sickle cell disease: results of a U.S. survey study
Source: Hematology. Author manuscript; Available in PMC 2019 Dec 1. (PMC6349225; doi:10.1080/16078454.2018.1549801)
Supplement: Supplementary Tables 1-6 [file NIHMS1515453-supplement-Supplementary_Tables_1-6.docx]

**Supplemental Tables**

**Supplemental Table 1**: Whether sites had a hospital affiliated with the outpatient sickle cell center and the type of clinics they enrolled participants from (pediatric, adult, or both)

|  |  | Had hospital affiliated with the outpatient sickle cell center | Pediatric or adult clinic (model of care) |
| --- | --- | --- | --- |
| Site | Midwest region:  Cincinnati | Yes | Pediatric |
|  | Chicago | Yes | Pediatric |
|  | Western region:  Oakland  Mid-South region: | Yes | Adult |
|  |  |  |  |
|  | St. Jude | Yes | Pediatric |
|  | UTHSC^a^ | Yes | Adult |
|  | Vanderbilt | Yes | Pediatric and Adult |

^a^UTHSC = University of Tennessee Health Science Center

|  |  | | **Supplementary Table 2: Socio-demographics for survey participants with sickle cell disease** | | | | |
| --- | --- | --- | --- | --- | --- | --- | --- |
| Variable | |  | | Adults  (N=167) | Children^a^ (N=364) | Combined (N=531) |  |
| Age | | Years (Median, Range) | | 28.0 (18.0-70.0) | 11.0 (0.0-36.0) | 14.0 (0.0-70.0) |  |
| Sex | | Male | | 71 (42.5%) | 174 (47.8%) | 245 (46.1%) |  |
|  |  | Female | | 96 (57.5%) | 190 (52.2%) | 286 (53.9%) |  |
| Race/Ethnicity | | Black, African American, African, or Afro-Caribbean | | 161 (96.4%) | 355 (97.5%) | 516 (97.2%) |  |
|  |  | Hispanic, Latino, or Spanish origin | | 2 (1.2%) | 8 (2.2%) | 10 (1.9%) |  |
|  |  | Some other race or origin | | 8 (4.8%) | 11 (3.0%) | 19 (3.6%) |  |
| Highest degree or level of school completed | | High school graduate or less | | 66 (39.5%) | 152 (41.8%) | 218 (41.1%) |  |
|  |  | Some college or beyond | | 98 (58.7%) | 140 (38.5%) | 238 (44.8%) |  |
| Household size | | Median (Range) | | 3 (1–14) | 4 (1-8) | 4 (1-14) |  |
| Marital status | | Married/Living together  Unmarried | | 46 (27.5%)  121 (72.5%) | 114 (31.3%)  250 (68.7%) | 160 (30.1%)  371 (69.9%) |  |
| Spirituality/Religiosity | | Very  Fairly  Slightly/Not at All | | 62 (37.1%)  76 (45.5%)  33 (19.8%) | 164 (45.1%)  113 (31.0%)  70 (19.2%) | 226 (42.6%)  189 (35.6%)  103 (19.4%) |  |
| Difficulty paying monthly bills | | Not very/Not at all  Somewhat/Very | | 94 (56.3%)  73 (43.7%) | 196 (53.8%)  168 (46.2%) | 290 (54.6%)  241 (45.4%) |  |
| Site | | Midwest region:  Cincinnati | | 0 (0.0%) | 51 (14.0%) | 51 (9.6%) |  |
|  | | Chicago | | 0 (0.0%) | 101 (27.7%) | 101 (19.0%) |  |
|  | | Western region:  Oakland  Mid-South region: | | 44 (26.3%) | 0 (0.0%) | 44 (8.3%) |  |
|  | | St. Jude | | 0 (0.0%) | 161 (44.2%) | 161 (30.3%) |  |
|  | | UTHSC^c^ | | 40 (24.0%) | 0 (0.0%) | 40 (7.5%) |  |
|  | | Vanderbilt | | 83 (49.7%) | 51 (14.0%) | 134 (25.2%) |  |

^a^Caregivers were asked to report for their children under 18 years

^b^Percentages may not add up to 100% because of missing data

^c^UTHSC = University of Tennessee Health Science Center

| **Supplementary Table 3: Scores on standardized measures for the survey participants (n = 531) with sickle cell disease** | | | | | | |
| --- | --- | --- | --- | --- | --- | --- |
| **Measure** |  | Adults  (N=167) | | | Children^a^ (N=364) | Combined (N=531) |
| **Patient Health Questionnaire (PHQ-2; mean/SD)** |  | | | 1.53 (1.61) | 0.87 (1.26) | 1.08 (1.41) |
| **ENRICHD Social Support Instrument (ESSI) (n/%)** | Poor | | 43 (25.7%) | | 35 (9.6%) | 78 (14.7%) |
|  | Good | | 134 (80.2%) | | 330 (90.7%) | 464 (87.4%) |
| **Brief Health Literacy Screening (n/%)** | Poor | | 47 (28.1%) | | 89 (24.5%) | 136 (25.6%) |
|  | Good | | 130 (77.8%) | | 276 (75.8%) | 406 (76.5%) |

^a^Caregivers were asked to report for their children under 18 years

^b^Percentages may not add up to 100% because of missing data

| **Supplementary Table 4: Questions about admissions, readmissions, and appointment keeping (n = 531)** | | | |  |  |
| --- | --- | --- | --- | --- | --- |
| **Question** | | Response | Adults  (N=167) | Children^a^ (N=364) | Combined (N=531) |
| **Admissions** | |  |  |  |  |
| Have you (or your child) been admitted to the hospital within the last year? | | No | 47 (28.1%) | 208 (57.1%) | 255 (48.0%) |
|  |  | Yes | 120 (71.9%) | 156 (42.9%) | 276 (52.0%) |
| I was unable to get the medication(s) I (or my child) needed. | | No | 79 (65.8%) | 146 (93.6%) | 225 (127.8%) |
|  |  | Yes | 36 (30%) | 10 (6.4%) | 46 (26.1%) |
| I did not feel that my (or my childs) medication was working. | | No | 60 (50%) | 112 (71.8%) | 172 (97.7%) |
|  |  | Yes | 55 (45.8%) | 44 (28.2%) | 99 (56.3%) |
| I did not have a good understanding of how often and how much of each medication I (or my child) needed. | | No | 104 (86.7%) | 148 (94.9%) | 252 (143.2%) |
|  |  | Yes | 10 (8.3%) | 8 (5.1%) | 18 (10.2%) |
| I did not have a good understanding of the major side effects of my (or my childs) medications. | | No | 106 (88.3%) | 146 (93.6%) | 252 (143.2%) |
|  |  | Yes | 10 (8.3%) | 10 (6.4%) | 20 (11.4%) |
| My (or my childs) pain was not able to be controlled at home. | | No | 17 (14.2%) | 48 (30.8%) | 65 (36.9%) |
|  |  | Yes | 102 (85%) | 108 (69.2%) | 210 (119.3%) |
| I did not have all the information I needed to take care of my (or my childs) illness at home. | | No | 98 (81.7%) | 145 (92.9%) | 243 (138.1%) |
|  |  | Yes | 17 (14.2%) | 11 (7.1%) | 28 (15.9%) |
| I did not understand which warning signs and symptoms meant I should call my (or my childs) healthcare provider. | | No | 100 (83.3%) | 145 (92.9%) | 245 (139.2%) |
|  |  | Yes | 15 (12.5%) | 11 (7.1%) | 26 (14.8%) |
| I (or my child) had an illness unrelated to their sickle cell disease (e.g., asthma). | | No | 71 (59.2%) | 110 (70.5%) | 181 (102.8%) |
|  |  | Yes | 43 (35.8%) | 46 (29.5%) | 89 (50.6%) |
| I (or my child) needed to get fluids or blood transfusion | | No | 35 (29.2%) | 65 (41.7%) | 100 (56.8%) |
|  |  | Yes | 80 (66.7%) | 91 (58.3%) | 171 (97.2%) |
| I (or my child) had a fever | | No | 74 (61.7%) | 70 (44.9%) | 144 (81.8%) |
|  |  | Yes | 42 (35%) | 86 (55.1%) | 128 (72.7%) |
| **Readmissions** | |  |  |  |  |
| Have you (or your child) been admitted to the hospital twice in the same month within the last year? | | No | 113 (67.7%) | 331 (90.9%) | 444 (83.6%) |
|  |  | Yes | 54 (32.3%) | 33 (9.1%) | 87 (16.4%) |
| I was unable to get the medication(s) I (or my child) needed. | | No | 27 (50%) | 30 (90.9%) | 57 (65.5%) |
|  |  | Yes | 21 (38.9%) | 3 (9.1%) | 24 (27.6%) |
| I did not feel that my (or my childs) medication was working. | | No | 19 (35.2%) | 23 (69.7%) | 42 (48.3%) |
|  |  | Yes | 33 (61.1%) | 10 (30.3%) | 43 (49.4%) |
| I did not have a good understanding of how often and how much of each medication I (or my child) needed. | | No | 45 (83.3%) | 31 (93.9%) | 76 (87.4%) |
|  |  | Yes | 3 (5.6%) | 2 (6.1%) | 5 (5.7%) |
| I did not have a good understanding of the major side effects of my (or my childs) medications. | | No | 42 (77.8%) | 30 (90.9%) | 72 (82.8%) |
|  |  | Yes | 5 (9.3%) | 3 (9.1%) | 8 (9.2%) |
| My (or my childs) pain was not able to be controlled at home. | | No | 7 (13%) | 10 (30.3%) | 17 (19.5%) |
|  |  | Yes | 45 (83.3%) | 23 (69.7%) | 68 (78.2%) |
| I did not have all the information I needed to manage my (or my childs) illness at home. | | No | 40 (74.1%) | 27 (81.8%) | 67 (77%) |
|  |  | Yes | 9 (16.7%) | 6 (18.2%) | 15 (17.2%) |
| I did not understand which warning signs and symptoms meant I should call my (or my childs) healthcare provider. | | No | 42 (77.8%) | 28 (84.8%) | 70 (80.5%) |
|  |  | Yes | 6 (11.1%) | 5 (15.2%) | 11 (12.6%) |
| I (or my child) had an illness unrelated to their sickle cell disease (e.g., asthma). | | No | 30 (55.6%) | 26 (78.8%) | 56 (64.4%) |
|  |  | Yes | 18 (33.3%) | 7 (21.2%) | 25 (28.7%) |
| I (or my child) was not healthy enough to leave the hospital during the first stay. | | No | 17 (31.5%) | 23 (69.7%) | 40 (46%) |
|  |  | Yes | 32 (59.3%) | 10 (30.3%) | 42 (48.3%) |
| I (or my child) had a fever | | No | 29 (53.7%) | 14 (42.4%) | 43 (49.4%) |
|  |  | Yes | 20 (37%) | 18 (54.5%) | 38 (43.7%) |
| **Missed appointments** | |  |  |  |  |
| Have you missed an appointment for any reason over the past year? | | No | 15 (9.0%) | 99 (27.2%) | 114 (21.5%) |
|  |  | Yes | 152 (91.0%) | 265 (72.8%) | 417 (78.5%) |
| Reasons you missed an appointment | | I forgot I had an appointment  The appointment was at a time that didn’t work for me  My health impacted my ability to make the appointment  I did not know I had an appointment | 60 (39.4%)  43 (28.3%)  36 (23.7%)  30 (19.7%) | 59 (22.3%)  51 (19.2%)  13 (4.9%)  29 (10.9%) | 119 (28.5%)  94 (22.5%)  49 (11.8%)  59 (14.1%) |
|  |  | I didn’t have a way to get to the appointment | 36 (23.7%) | 57 (21.5%) | 93 (22.3%) |
|  |  | |  |  |  |

^a^Caregivers were asked to report for their children under 18 years

^b^Percentages may not add up to 100% because of missing data

| **Supplementary Table 5. Logistic regression model: risk factors for hospitalizations and readmissions** |
| --- |
| **Hospitalizations** |
|  |

| **Combined Model** | **(n=531)** |  |  |  |
| --- | --- | --- | --- | --- |
| **Variable** |  | **Odds Ratio** | **95% CI** | **Pr(>\|z\|)** |
| (Intercept) |  | 1.43 | (0.62,3.28) | 0.402 |
| Age Group | Pediatric model of care | 0.27 | (0.17,0.42) | <0.001* |
| Sex | Female | 1.16 | (0.77,1.73) | 0.481 |
| Education: | Some college or more | 1.17 | (0.76,1.78) | 0.476 |
| PHQ score |  | 1.36 | (1.16,1.6) | <0.001* |
| Ability to pay bills | Very or somewhat difficult | 1.46 | (0.96,2.22) | 0.074† |
| Literacy | High | 0.8 | (0.48,1.31) | 0.371 |
| Spirituality | Very spiritual | 0.76 | (0.5,1.15) | 0.2 |
| Social Support | High | 1.35 | (0.73,2.5) | 0.345 |
| **Adult Model** | **(n=167)** |  |  |  |
| **Variable** |  | **Odds Ratio** | **95% CI** | **Pr(>\|z\|)** |
| (Intercept) |  | 2.6 | (0.39,17.16) | 0.321 |
| Age |  | 0.96 | (0.93,0.99) | 0.024* |
| Sex | Female | 0.69 | (0.3,1.58) | 0.383 |
| Education: | Some college or more | 1.58 | (0.69,3.6) | 0.276 |
| PHQ score |  | 1.63 | (1.18,2.25) | 0.003* |
| Ability to pay bills | Very or somewhat difficult | 4.43 | (1.69,11.58) | 0.002* |
| Literacy | High | 1.8 | (0.67,4.84) | 0.244 |
| Spirituality | Very spiritual | 0.41 | (0.16,1.03) | 0.057† |
| Social Support | High | 1.49 | (0.5,4.43) | 0.477 |
| **Model about Children** | (**n=364)** |  |  |  |
| **Variable** |  | **Odds Ratio** | **95% CI** | **Pr(>\|z\|)** |
| (Intercept) |  | 0.45 | (0.14,1.42) | 0.172 |
| Age |  | 1 | (0.96,1.05) | 0.881 |
| Sex | Female | 1.21 | (0.74,1.96) | 0.443 |
| Education: | Some college or more | 1.13 | (0.68,1.89) | 0.636 |
| PHQ score |  | 1.26 | (1.03,1.53) | 0.024* |
| Ability to pay bills | Very or somewhat difficult | 1.07 | (0.65,1.75) | 0.797 |
| Literacy | High | 0.67 | (0.36,1.23) | 0.193 |
| Spirituality | Very spiritual | 0.85 | (0.52,1.4) | 0.528 |
| Social Support | High | 1.5 | (0.65,3.47) | 0.34 |
|  |  |  |  |  |

| **Readmissions** |  |  |  |  | |
| --- | --- | --- | --- | --- | --- |
| **Combined Model** | **(n=531)** |  |  |  | |
| **Variable** |  | **Odds Ratio** | **95% CI** | **Pr(>\|z\|)** | |
| (Intercept) |  | 0.31 | (0.11,0.86) | 0.025 | |
| Age Group | Pediatric model of care | 0.21 | (0.12,0.37) | <0.001* | |
| Sex | Female | 1.38 | (0.79,2.42) | 0.254 | |
| Education: | Some college or more | 1 | (0.57,1.77) | 0.989 | |
| PHQ score |  | 1.24 | (1.04,1.49) | 0.019* | |
| Ability to pay bills | Very or somewhat difficult | 2.4 | (1.36,4.24) | 0.003* | |
| Literacy | High | 1.16 | (0.6,2.25) | 0.66 | |
| Spirituality | Very spiritual | 0.57 | (0.33,1) | 0.049* | |
| Social Support | High | 0.67 | (0.33,1.35) | 0.263 | |
| **Adult Model** | **(n=167)** |  |  |  | |
| **Variable** |  | **Odds Ratio** | **95% CI** | **Pr(>\|z\|)** | |
| (Intercept) |  | 1.76 | (0.28,11.01) | 0.543 | |
| Age |  | 0.96 | (0.92,0.99) | 0.018* | |
| Sex | Female | 1.02 | (0.44,2.36) | 0.967 | |
| Education: | Some college or more | 1.27 | (0.57,2.84) | 0.555 | |
| PHQ score |  | 1.26 | (0.99,1.61) | 0.062† | |
| Ability to pay bills | Very or somewhat difficult | 4.18 | (1.85,9.44) | 0.001* | |
| Literacy | High | 1.31 | (0.51,3.35) | 0.573 | |
| Spirituality | Very spiritual | 0.26 | (0.11,0.61) | 0.002* | |
| Social Support | High | 0.5 | (0.2,1.25) | 0.136 | |
| **Model about**  **Children** | (**n=364)** |  |  |  | |
| **Variable** |  | **Odds Ratio** | **95% CI** | **Pr(>\|z\|)** | |
| (Intercept) |  | 0.02 | (0,0.16) | 0 | |
| Age |  | 1.04 | (0.96,1.12) | 0.359 | |
| Sex | Female | 1.63 | (0.68,3.9) | 0.273 | |
| Education: | Some college or more | 0.85 | (0.35,2.08) | 0.723 | |
| PHQ score |  | 1.24 | (0.91,1.68) | 0.167 | |
| Ability to pay bills | Very or somewhat difficult | 1.25 | (0.53,2.96) | 0.609 | |
| Literacy | High | 1.47 | (0.48,4.48) | 0.496 | |
| Spirituality | Very spiritual | 1.11 | (0.46,2.65) | 0.817 | |
| Social Support | High | 1.62 | (0.34,7.79) | 0.55 | |
| \| UTHSC: University of Tennessee Health Science Center; CHO: Children’s Hospital Oakland; CCHMC: Cincinnati Children’s Hospital Medical Center  * p <0.05; † p < 0.1 \| \| --- \| | | | | | |
|  |  |  |  | |  |

| \| **Supplementary Table 6. Relation between reasons for missing clinic appointments and hospital admissions and readmissions** \| \| --- \|   **Hospital admission in the past year** | | |
| --- | --- | --- | --- |
| Combined |  |  |
| Have you missed an appointment for any reason | No | Yes |
| No admission | 66 (57.9%) | 189 (45.3%) |
| Admission | 48 (42.1%) | 228 (54.7%) |
| P-value | 0.02 |  |
| OR (95% CI) | 1.66 (1.07-2.58) |  |
| Adults |  |  |
| Have you missed an appointment for any reason | No | Yes |
| No admission | 8 (53.3%) | 39 (25.7%) |
| Admission | 7 (46.7%) | 113 (74.3%) |
| P-value | 0.03 |  |
| OR (95% CI) | 3.28 (0.97-11.40) |  |
| Children^a^ |  |  |
| Have you missed an appointment for any reason | No | Yes |
| No admission | 58 (58.6%) | 150 (56.6%) |
| Admission | 41 (41.4%) | 115 (43.4%) |
| P-value | 0.812 |  |
| OR (95% CI) | 1.08 (0.66-1.78) |  |
|  | | |
| **Readmission in the past year** | | |
| Combined |  |  |
| Have you missed an appointment for any reason | No | Yes |
| No readmission | 105 (92.1%) | 339 (81.3%) |
| Readmission | 9 (7.9%) | 78 (18.7%) |
| P-value | 0.004 |  |
| OR (95% CI) | 2.68 (1.28-6.29) |  |
| Adults |  |  |
| Have you missed an appointment for any reason | No | Yes |
| No readmission | 14 (93.3%) | 99 (69.7%) |
| Readmission | 1 (6.7%) | 53 (30.3%) |
| P-value | 0.039 |  |
| OR (95% CI) | 7.44 (1.07-322.34) |  |
| Children^a^ |  |  |
| Have you missed an appointment for any reason | No | Yes |
| No readmission | 91 (91.9%) | 214 (91.1%) |
| Readmission | 8 (8.1%) | 21 (8.9%) |
| P-value | 0.838 |  |
| OR (95% CI) | 1.18 (0.50-3.15) |  |

^a^Caregivers were asked to report for their children under 18 years
